# Estimating Sizes of Key Populations at the National Level: Considerations for Study Design and Analysis

**DOI:** 10.1097/EDE.0000000000000906

**Published:** 2018-09-28

**Authors:** Jessie K. Edwards, Sarah Hileman, Yeycy Donastorg, Sabrina Zadrozny, Stefan Baral, James R. Hargreaves, Elizabeth Fearon, Jinkou Zhao, Abhirup Datta, Sharon S. Weir

**Affiliations:** From the a Department of Epidemiology, University of North Carolina at Chapel Hill: Chapel Hill, NC; b Carolina Population Center, University of North Carolina at Chapel Hill: Chapel Hill, NC; c Instituto Dermatológico y Cirugía de Piel Dr. Huberto Bogaert Díaz; d Department of Epidemiology, Johns Hopkins School of Public Health, Baltimore, MD; e Department of Social and Environmental Health Research, London School of Hygiene and Tropical Medicine: London, UK; f The Global Fund, Geneva, Switzerland; g Department of Biostatistics, Johns Hopkins School of Public Health, Baltimore, MD.

**Keywords:** HIV, Sex workers, Sexual and gender minorities, Epidemiologic methods

## Abstract

**Background:**

National estimates of the sizes of key populations, including female sex workers, men who have sex with men, and transgender women are critical to inform national and international responses to the HIV pandemic. However, epidemiologic studies typically provide size estimates for only limited high priority geographic areas. This article illustrates a two-stage approach to obtain a national key population size estimate in the Dominican Republic using available estimates and publicly available contextual information.

**Methods:**

Available estimates of key population size in priority areas were augmented with targeted additional data collection in other areas. To combine information from data collected at each stage, we used statistical methods for handling missing data, including inverse probability weights, multiple imputation, and augmented inverse probability weights.

**Results:**

Using the augmented inverse probability weighting approach, which provides some protection against parametric model misspecification, we estimated that 3.7% (95% CI = 2.9, 4.7) of the total population of women in the Dominican Republic between the ages of 15 and 49 years were engaged in sex work, 1.2% (95% CI = 1.1, 1.3) of men aged 15–49 had sex with other men, and 0.19% (95% CI = 0.17, 0.21) of people assigned the male sex at birth were transgender.

**Conclusions:**

Viewing the size estimation of key populations as a missing data problem provides a framework for articulating and evaluating the assumptions necessary to obtain a national size estimate. In addition, this paradigm allows use of methods for missing data familiar to epidemiologists.

In many countries, the HIV epidemic is concentrated among key populations, including sex workers, men who have sex with men, people who inject drugs, and transgender women.^1,2^ Even in countries with generalized HIV epidemics, key populations have disproportionate risks for the acquisition and transmission of HIV that include biologic, network, and structural risks. National estimates of the sizes of key populations are critical to inform national and international responses to the HIV pandemic, including prioritization of public health programs, resource allocation, intervention planning, and evaluation.^3^

However, key population size estimates are typically incomplete, often available only for towns or areas included in epidemiologic studies or surveillance sites.^4^ These subnational size estimates are typically derived from programmatic mapping or from sample surveys using Time Location Sampling^5–7^ or Respondent Driven Sampling^8–10^ that are most effectively conducted in limited geographic areas. Moreover, the data collection activities required to obtain reasonable estimates of the sizes of key populations are resource intensive, particularly when the population of interest is hidden, stigmatized, or legally criminalized, such as sex workers, people who inject drugs, and men who have sex with men. Thus, available estimates tend to be constrained and are often derived from sites selected on the basis of perceived need rather than with national representativeness in mind.^11^

Despite these challenges, there is increasing demand for national size estimates to guide HIV-related decision making and global reporting.^2,12^ Existing international guidelines^11,13,14^ suggest a range of approaches to obtain national estimates from incomplete data, including (1) applying the average prevalence of a given key population to all areas without a direct estimate; (2) applying the average prevalence of a given key population from a certain stratum of an important variable (e.g., population density) to areas without a direct estimate within that stratum; or (3) matching areas without estimates to areas with direct estimates that “are most similar in terms of HIV risk.”^11^

However, these ad-hoc approaches rely on hidden assumptions, and current guidelines provide little guidance on how to select between the proposed methods or choose important covariates for matching or stratification. Here, we demonstrate how the need for a national key population size estimate maps on to a standard missing data problem in epidemiology and how modern epidemiologic theory and methods developed to handle missing data can guide analyses in this setting. We illustrate this approach to estimate the sizes of key populations at the national level using an example from the Dominican Republic (DR).

To improve HIV-related services for key populations in the DR, a 2014 study obtained estimates of the sizes of key populations in priority areas.^15^ This article details how epidemiologic methods for missing data and targeted additional data collection were used to develop national key population size estimates. Because data collection efforts were targeted to areas at high perceived risk, we hypothesized that using data from the 2014 study alone would overestimate the national sizes of the key population groups.

## METHODS

Throughout this article, we will refer to the estimated sizes of key populations from specific data collection activities in defined geographic regions as *direct estimates.* In this example, as in many countries, direct estimates were obtained from areas chosen for programmatic planning purposes, rather than to achieve a representative sample of areas within the country.

We focus on describing methods and assumptions that can be used to generalize results from areas with direct estimates of the parameters of interest to the national level. Specifically, the parameters of interest were point prevalences in 2016 corresponding to (1) the proportion of the adult female population (ages 15–49) in the DR engaged in sex work; (2) the proportion of the adult male population (ages 15–49) who engage in sex with another man; and (3) the proportion of people assigned the male sex at birth (ages 15–49) who were transgender women. This article describes a two-stage sampling approach and analytic methods to estimate these parameters.

In the two-stage approach, direct estimates for a subset of areas sampled for programmatic planning purposes (stage 1) were augmented by direct estimates from a smaller random sample of areas (stage 2) and contextual data available for all areas. We compare analytic strategies to analyze the resulting data using inverse probability weights, multiple imputation (MI), and augmented inverse probability weighting.

### Assumptions for Missing Data

We view the need for a national population size estimate as a missing data problem in which data are missing for geographic areas without direct estimates. As such, we rely on the standard assumptions for inference in the presence of missing data, namely that areas with and without missing data are exchangeable. Exchangeability implies that the expected proportion of men or women who fall into each key population is the same in areas with and without direct estimates.^16–18^ However, when at least some areas are purposively selected based on perceived risk, as in stage 1 of this example, the proportion falling within a key population may systematically differ between sampled and nonsampled areas.

In this case, we may relax the exchangeability assumption to be conditional on contextual covariates 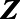, such that we assume exchangeability only within strata of these covariates, or that the key population size is independent of sampling into the study, given 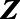.^19–21^ However, relaxing the exchangeability assumption to be conditional on the context 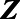 requires that we additionally assume that at least some areas are sampled within all levels of 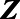. This is also known as the positivity assumption.^22^ The sections that follow illustrate how these assumptions were used to guide our study design and analysis.

### Stage 1: Direct Size Estimates from a Program Planning Survey

The DR is divided into 154 municipalities nested within 31 provinces. Direct estimates of the sizes of key populations were available from a 2014 Priorities for Local AIDS Control Efforts (PLACE) study conducted in 30 municipalities randomly sampled from six areas perceived by national stakeholders to be at high risk of HIV transmission.^15^ These municipalities are highlighted in panel A of the Figure. All other municipalities originally had no direct estimates of the parameters of interest. Full details of the 2014 PLACE study have been previously published.^15^ Briefly, the purpose of the PLACE 2014 study was to describe the characteristics, access to HIV prevention services, and risk behaviors among people socializing in public places, including key populations. As part of its mandate, the study produced estimates of the sizes of the populations of sex workers, men who have sex with men (MSM), and transgender women for the selected municipalities. The Comisión Nacional de Bioética en Salud in the DR and the University of North Carolina institutional review board approved all study protocols.

**FIGURE.**
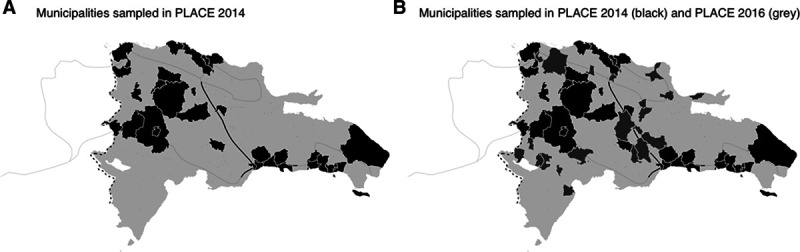
Map of the Dominican Republic with municipalities purposively sampled in 2014 in black (panel A) and with randomly sampled municipalities added in gray (panel B).

### Stage 2: Direct Size Estimates from a Sample of Municipalities

The sampling frame for the 30 municipalities selected for direct estimates in stage 1 was limited to perceived high burden areas. Accordingly, municipalities with and without direct estimates in stage 1: (1) were not likely to be unconditionally exchangeable; and (2) may have been exchangeable within levels of important contextual variables, but it is likely that not all levels of these variables were represented in the sample (i.e., the positivity assumption was violated).

Therefore, we obtained additional direct estimates of the sizes of key populations through a 2016 PLACE study conducted in 20 additional municipalities. Panel B of Figure displays all municipalities sampled during either stage 1 or stage 2 data collection activities. More information about the 2014 and 2016 PLACE studies and direct estimates from all sampled municipalities, can be found in eAppendices 1 and 2; http://links.lww.com/EDE/B400.

### Contextual Information

Direct estimates of key population sizes were available only for municipalities with data collection activities in 2014 or 2016, but municipal-level contextual information was available for all municipalities. Contextual information came from publicly available sources that provided insight into how sampled municipalities differed from nonsampled municipalities with regard to variables that predicted the sizes of the key populations of interest.

Key stakeholders in the HIV research, treatment, and advocacy communities in the DR identified important contextual variables using diagrams,^21,23^ namely, those variables that were both associated with sampling and the sizes of each key population. Information on contextual variables was obtained from the Oficina Nacional de Estadística, stakeholder knowledge, and the DR 2013 Demographic and Health Surveys (DHS).^24^ From Oficina Nacional de Estadística, we retrieved information on total population density, the joint distribution of age and sex, the proportion of the population of Haitian descent, and the proportion living in poverty for each municipality. Stakeholders from the Ministry of Health provided input on the presence of tourist areas, borders, and ports, and the count of universities within each municipality; this information was verified by the study team using geographic databases.

We used data from the 2013 DHS to estimate the overall HIV prevalence, average number of years of education among women, and proportion of female adolescents who were pregnant in each municipality. Because the DHS is designed to generalize to the DHS region level, rather than the municipal level, we interpolated each of the above indicators between DHS clusters for each cell on a fine grid overlaid on the country.^25^ Values were interpolated only for grid cells within the convex hull determined by the cluster locations using the R package *akima*,^26^ and summarized by taking the average within grid cells falling within each municipality. Contextual variables contained no missing data.

### Statistical Methods

Let the number of municipalities 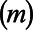 be indexed as 
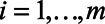
 and 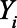 represent the count of the key population of interest in municipality 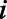. 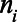 is the population in municipality 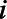
that could be part of the key population of interest if they met the defining criteria (i.e., for female sex workers, 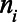 is the total number of women ages 15–49 and for MSM and transgender women, 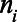 is the number of people assigned male sex at birth ages 15–49). For each of the three parameters of interest, we represent this proportion in each municipality as 
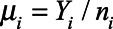
 and at the national level as 
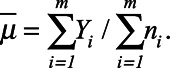
 For municipalities without direct estimates, 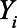, and therefore 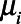, are missing. We assume the parameters of interest are stable from 2014 to 2016 such that data from both data collection efforts may be used to estimate a single set of key population sizes.

Under the assumption that the proportion of the population falling within each key population of interest is the same (i.e., *exchangeable*) between sampled and nonsampled municipalities, 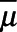 could be consistently estimated as the proportion classified as a member of that key population in the sampled municipalities (“complete cases”) only. Using a complete case approach, we estimated 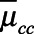 as 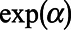 in the Poisson regression model 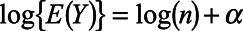 fit to the sampled municipalities.

We next relaxed the exchangeability assumption to be conditional on a set of contextual variables 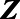 that both predicted the sizes of the key populations and differed between sampled and nonsampled areas. Because the set of contextual variables affecting key population size varies by key population, stakeholders selected a separate set of covariates for female sex workers, MSM, and transgender women populations. All models included population density, the proportion of people living in poverty or extreme poverty, presence of tourism, and HIV prevalence among the general population. For female sex workers, 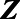 additionally included the proportion of female adolescents pregnant at the time of the DHS survey, the mean number of years of education among women, and presence of an international border or port. For MSM and transgender populations, 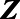 additionally included the presence of universities in the municipality.

We explored three analytic approaches to relax the exchangeability assumption. First, we used an inverse probability of sampling weighted (IPSW) approach in which sampled municipalities included in the Poisson model used in the complete case approach were up-weighted based on 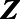 to represent all municipalities in the country. Weights for each municipality, denoted by 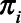, were defined as the inverse probability that a municipality was sampled, conditional on 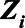, or 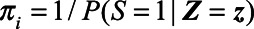. The conditional probability of sampling in the denominator was estimated using the logistic regression 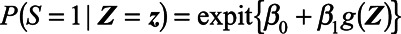, where 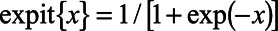 and 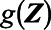 indicates that variables in 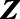 were modeled using flexible functional forms (e.g., restricted quadratic splines^27^). 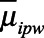 was estimated as 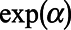 in the weighted Poisson model 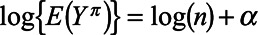, where the superscript 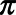 indicates that sampled municipalities were weighted by 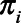. Ninety-five percentage confidence intervals (CIs) were constructed using the robust sandwich variance estimator.^28^

Next, we used MI^29,30^ to impute the number of people in each key population in municipalities without direct estimates. We first fit a Poisson regression model for the count of each key population in municipalities with direct estimates, conditional on 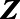, 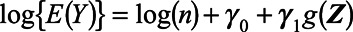. We then drew a set of regression coefficients for each of 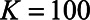 imputations from the posterior distribution of the parameters 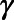. We assumed parameters followed a multivariate normal distribution with mean vector (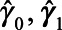) and covariance matrix 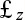. We created a new variable 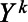 to represent the count of the key population of interest in imputation 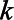. For municipalities with direct estimates, 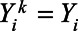 for all imputations. For municipalities without direct estimates, 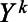 was imputed based on the regression coefficients 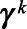 drawn for imputation 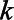, such that 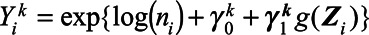.

Finally, we fit an analysis model in each imputed dataset and summarized across imputations. The analysis model was the Poisson regression model 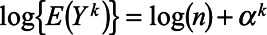, and the estimated proportion in each key population 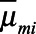 was 
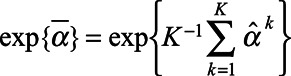
, where 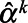 was the natural log of the proportion in each key population from the 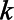
th imputed dataset. The variance for 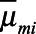 was given by Rubin’s rules^29^
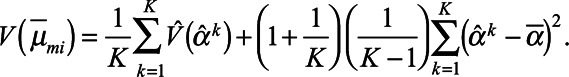


A third approach estimated 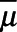 using an augmented IPSW approach. The standard IPSW approach relied on correct specification of the logistic regression model for the probability of being sampled into the study, while the MI approach relied on correct specification of the Poisson model for 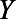 conditional on 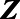. The augmented IPSW approach was designed to improve on the efficiency of the standard IPSW estimator and to yield a consistent estimate of 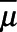 if the statistical specification of either the model for sampling or the model for the outcome were correct.^31,32^ Note that at least one of the models must include all variables needed for exchangeability between sampled and nonsampled municipalities and neither model may contain variables affected by sampling (e.g., mediators) or colliders.^33^ We implemented this approach using the “regression” augmented IPW estimator described by Robins et al.^34^ (and implemented by others; e.g.,^35^) designed to improve the performance of standard IPW estimators.

To implement this approach, we predicted *Y* by fitting the weighted Poisson regression model 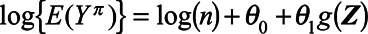, where the weights were the inverse probability of sampling described above. 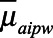 was estimated as 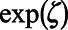 in the Poisson model for 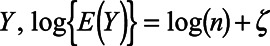, where 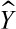 is the predicted count obtained using 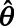. Ninety-five percentage CIs for 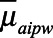 were constructed as 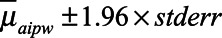, where the standard error was estimated as the standard deviation of 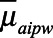 from 1,000 bootstrap samples of the original data.^36^

We explored the finite sample properties of the three analytic approaches to relax the exchangeability assumption using simulation experiments. Details on the simulation design and results can be found in the Appendix. SAS code to analyze a sample simulated dataset is provided in eAppendix 3; http://links.lww.com/EDE/B400.

## RESULTS

Overall, sampled municipalities had slightly lower HIV prevalence, higher population density, a lower proportion of people living in poverty, and a greater proportion of female adolescents pregnant at the time of the DHS survey than nonsampled municipalities (Table 1). The proportion of people of Haitian descent and the average number of years of education among the female population were similar between the groups, though sampled municipalities were more likely to have tourism, an international border or port, or a university than nonsampled municipalities. In the PLACE 2014 data, strata with low population density and/or a high proportion living in poverty had very few sampled municipalities (Table 2). In the 2016 sample and the union of the two datasets, all strata are represented.

**Table 1.**
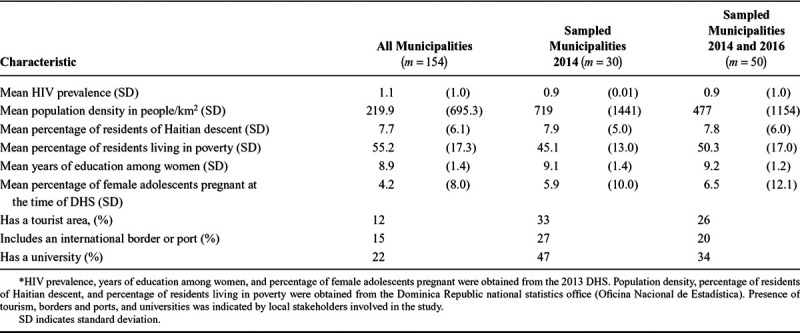
Characteristics* of the 154 Municipalities in the Dominican Republic and for the Municipalities Sampled for Direct Estimates of the Sizes of Key Populations in PLACE 2014 and the Combined PLACE 2014 and PLACE 2016 Sample

**Table 2.**
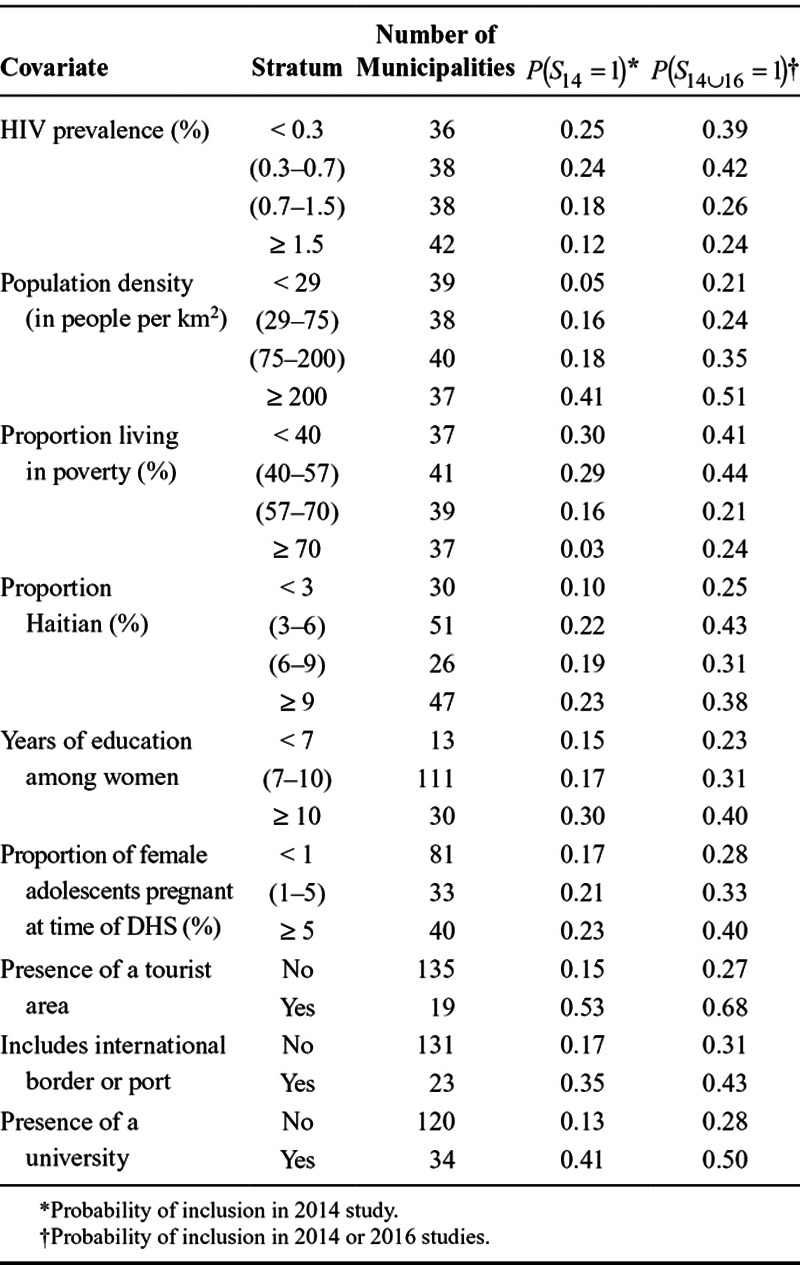
Assessing Positivity: Probability of sampling a municipality for Direct Estimates of Key Population Size in the PLACE 2014 Study or the Combined PLACE 2014 and PLACE 2016 Dataset Among 154 Municipalities in the Dominican Republic

For female sex worker and MSM populations, size estimates from the 2014 sample alone were lower than size estimates from the 2016 sample or the 2014 sample augmented with 2016 data (Table 3). In contrast, the estimated size of the transgender population was higher in the 2014 sample than in the augmented sample. The three approaches to account for differences between sampled and nonsampled municipalities yielded similar results. As expected, results from MI were most precise. Results from the augmented IPSW approach were similar to, though more precise than, the IPSW estimates. Using the augmented IPSW approach, we estimated that 3.7% (95% CI = 2.9, 4.7) of the total population of women between the ages of 15 and 49 was engaged in sex work. Using the same approach, we estimated that the MSM population was 1.2% (95% CI = 1.1, 1.3) and the population of transgender women was 0.19% (95% CI = 0.17, 0.21) of the total population between 15 and 49 assigned male sex at birth.

**Table 3.**
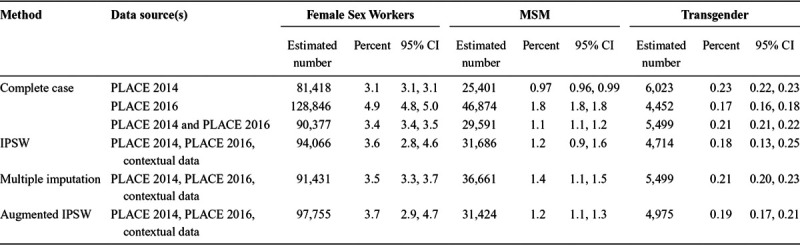
Sizes of Key Populations in the Dominican Republic Estimated Using PLACE 2014 Data Only, PLACE 2016 Data Only, and PLACE 2014, PLACE 2016, and Contextual Data

## DISCUSSION

The proposed two-stage approach produced estimates of the sizes of three key populations in the Dominican Republic under a set of well-defined assumptions. Estimates obtained using MI were most precise, but estimates from the augmented IPSW approach offered improved precision over the IPSW approach and were expected by theory to be more robust to model misspecification than either the MI or IPSW approaches. Based on results from the augmented IPSW analysis, there were 97,755 women (3.7% of women) engaged in sex work, 31,424 MSM (1.2% of men), and 4,975 transgender women (0.19% of people assigned male sex at birth) between the ages of 15 and 49 living in the DR in 2016. The estimated numbers of women engaged in sex work and MSM were higher under the proposed approach than would have been estimated by applying the crude proportion in each key population from the PLACE 2014 data alone (81,418 and 25,401, respectively), while the number of transgender women was slightly lower than would have been estimated from the PLACE 2014 data (6,023).

Taken together, these results highlight important considerations for the design and analysis of studies to estimate the sizes of key populations at the national level. Although data collected from purposively selected geographic areas for programmatic purposes can be (and often must be) leveraged to estimate the sizes of key populations,^4^ using such data to inform size estimates requires understanding the explicit or implicit sampling frame used. Knowledge of which segments of the population, based on demographics or location, are excluded systematically from the sampling frame is important to ensure these groups are represented through other sources of data or assumptions about the distributions of key populations in these groups.

Furthermore, generalizing the proportion of people in each key population to the national level requires collecting data on a minimally sufficient set of covariates conditional on which sampling is independent of key population size.^37,38^ Because the stakeholders who were involved in selecting the municipalities for PLACE 2014 identified the contextual variables that informed this selection, it is unlikely that we omitted important covariates. Here, we were able to gather values of these contextual variables using online publicly available data sources and stakeholder knowledge. In other settings, additional data collection activities may be required to measure these covariates. Note that, if size estimates are needed for *individual* municipalities currently missing data, one would need to model all predictors of key population size that vary by municipality, which may require more intensive assumptions (e.g., that all predictors of key population size were included) and data collection activities.

Consistently estimating key population size at the national level requires correct specification of any parametric models used. These models must include all variables needed for conditional exchangeability between sampled and nonsampled municipalities to hold. In the approaches outlined in this article, we used parametric models for sampling (the IPSW approach), key population size (the MI approach), and both (the augmented IPSW approach). These models may be difficult to specify because, while one would like to model all variables flexibly (e.g., using splines or nonparametric kernel smoothing techniques) and include interactions between variables, direct estimates are often based on data collected in few municipalities, making models with many parameters unstable. Bayesian techniques and frequentist shrinkage estimators offer approaches to reduce mean squared error by trading some bias to reduce the variance of resulting estimators.^39^ Indeed, recent work has outlined approaches to fit models in which the number of parameters approaches or exceeds the number of data points.^40^

The assumptions necessary to identify a national size estimate are analogous to assumptions necessary for quantitative generalizability in other epidemiologic applications,^19,37^ which can in turn be related to the assumptions necessary to make inference in the presence of missing data.^21^ Connecting the need for a national size estimate to the extensive literature on statistical approaches for missing data opens the door to a wide range of methods that can be adapted to suit the needs of each individual study.^21,30,32,35,41,42^

We expected that municipalities selected for data collection in 2014 due to high perceived risk of ongoing HIV transmission would have higher proportions of key populations than municipalities not sampled as part of this exercise. However, municipalities randomly sampled in 2016 had a higher proportion of women engaging in sex work and MSM than the municipalities purposively sampled in 2014, despite similar study protocols. This discrepancy has also been seen in other settings (e.g.^43^) and could have several causes. While areas identified by stakeholders as areas at high risk of ongoing HIV transmission likely had high counts of key populations, they were also areas with high population density, meaning that the proportion of the total population classified as part of a key population remained low. In addition, data collection activities in 2014 focused on urban municipalities, and therefore underrepresented rural areas where higher proportions of residents live in poverty. If sex work were associated with poverty, the 2014 data collection activities may have missed these pockets of sex work. Furthermore, changes in the distributions of key populations could have occurred during the 2-year gap between data collection activities or due to seasonal mobility of sex workers. Our findings underscore the value of objective confirmation of areas identified by stakeholders as high priority areas and the need for a rapid assessment tool to identify underserved clusters of key populations in areas outside priority program areas.

This study had several limitations. Although we assumed all direct estimates were measured without error, estimating the sizes of key populations is difficult, even at the local level, and depends on strong assumptions.^3^ Direct estimates in this study could be improved using results from a validation study employing a more rigorous measure of key population size or prior knowledge about the amount of measurement error present.^44–46^ Moreover, we assumed the values of direct estimates were *known* rather than estimated, as we did not take into account any uncertainty due to random error in the direct estimates, likely resulting in CIs that are too narrow. Although some methods to obtain direct size estimates produce standard 95% CIs, others provide bounds that take into account only possible systematic error, while still others provide no measure of variability at all. When extrapolating direct estimates with measures of random or systematic error, this error could be propagated through to the national estimate using a hierarchical modeling approach,^47^ resulting in wider intervals that illustrate the uncertainty present in both stages of the analysis.

Here, we have presented a framework for estimating the sizes of key populations at the national level. These estimates are in demand from national governments and international organizations, and ad-hoc approaches to combine existing data sources to produce such estimates may yield misleading results. This work offers a principled approach to obtaining a national population size estimate by articulating the assumptions needed, describing how to leverage various types of data, and illustrating three statistical techniques to obtain national estimates from incomplete data, thus improving the knowledge base that informs the public health response to the HIV pandemic.

## ACKNOWLEDGMENTS

We thank Rosa Sánchez of the Consejo Nacional para el VIH y el SIDA, the HIV Monitoring and Evaluation Technical Working Group, and representatives of nongovernmental organizations in the Dominican Republic for helpful comments during the design and implementation of this study.

## Figures and Tables

**Table:**
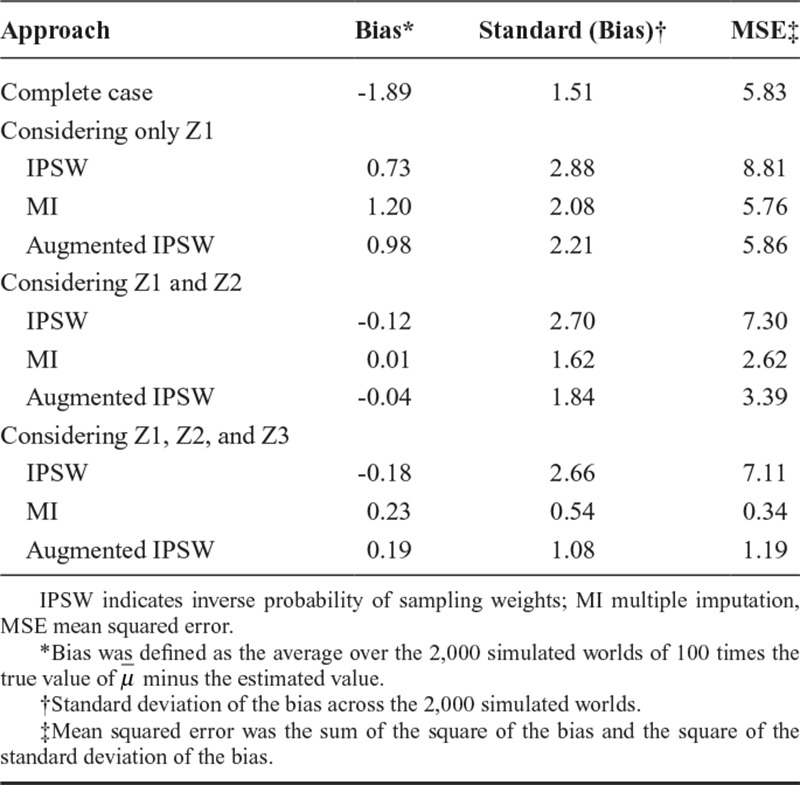
Appendix Table 1. Comparison of Bias, Precision, and Mean Squared Error Between Proposed Analytic Approaches in 2,000 Simulated Worlds

## APPENDIX. SIMULATION EXPERIMENTS

We conducted a series of simulation experiments to assess the finite sample performance of the proposed estimators (i.e., complete case analysis, inverse probability weighting, multiple imputation (MI), and augmented inverse probability weighting) used to analyze the two-stage data. For the purposes of the simulations, we assumed that data from both stages were available.

In each of 2000 simulated worlds, there were  to  units. In each unit, the proportion of interest was . The parameter of interest was the overall proportion . The purpose of the simulation experiment was to compare the bias and precision of each analytic approach to estimate the overall proportion  from data in which some units were missing information on .

Specifically, the simulated data consisted of 3 independent covariates:  and . Each covariate was a binary random variable with probabilities of 0.3, 0.75, and 0.2, respectively. Of the  units in each simulated dataset, approximately 30% had complete data , whereas 70% were missing information on .  and  predicted both sampling  and the outcome , while  predicted only  but was independent of sampling.

Each unit’s probability of sampling depended on  and  such that

And each unit’s proportion  depended on and  such that

And  was the number of successes drawn from  trials in a binomial distribution with probability .

In summary, units  were assigned variables , , and . In each simulated world, the true  was defined as . To compare the proposed approaches,  and  were set to missing where . In each simulated world,  was estimated using the complete case approach, the inverse probability weighting approach, the MI approach, and the augmented inverse probability weighting approach, as described in the text. Under each approach, we compared bias (defined as 100 times the average difference between the true value and the estimated value across the 2,000 simulated worlds), precision (defined as the standard deviation of the bias in the 2,000 simulated worlds), and mean squared error (the sum of the square of the bias and the square of the standard deviation of the bias).

Results are summarized in Appendix Table 1. The average true value of  was 5.8%. The complete case approach produced an estimate with substantial downward bias. When only Z1 was considered in the IPSW, MI, and augmented IPSW approaches, these approaches also produced biased results. However, adding Z2 reduced bias and improved precision under all approaches. When Z1 and Z2 were both considered, all approaches produced results with little bias. The MI approach was most precise followed by the augmented IPSW approach and then the IPSW approach. When Z3 was considered in addition to Z1 and Z3, results were slightly more precise, but bias was not substantially reduced for any approach (and actually increased marginally for IPSW and augmented IPSW approaches). This supports our assertion that one need not measure or include predictors of the outcome that are not associated with sampling to use the proposed approaches, though adding the additional predictor of the outcome did decrease mean squared error.
